# Overview and Recent Advances in Bioassays to Evaluate the Potential of Entomopathogenic Fungi Against Ambrosia Beetles

**DOI:** 10.3390/insects16060615

**Published:** 2025-06-10

**Authors:** Jesús Enrique Castrejón-Antonio, Patricia Tamez-Guerra

**Affiliations:** 1 Facultad de Ciencias Biológicas y Agropecuarias, Universidad de Colima, Autopista Colima-Manzanillo km 40, La Estación, Tecomán 28930, Colima, Mexico; 2 Facultad de Ciencias Biológicas, Departamento de Microbiología e Inmunología, Universidad Autónoma de Nuevo León, Av. Pedro de Alba S/N, Cd. Universitaria, San Nicolás de los Garza 66455, Nuevo León, Mexico; patricia.tamezgr@uanl.edu.mx

**Keywords:** bark beetles, biological control, inoculation, formulation, Scolytinae, pest management programs

## Abstract

Ambrosia beetles, known for their symbiotic relationship with fungi cultivated within the tissues of host trees, have become significant pests, particularly when they serve as vectors for pathogenic fungi such as *Raffaelea lauricola*. Given the regulatory and environmental constraints for chemical application as a tool for their control, entomopathogenic fungi (EPF) represent a promising pest management alternative. This review presents an overview of bioassays assessing the pathogenicity and virulence of EPF against ambrosia beetles. Most studies have been performed in vivo (artificial diet) under laboratory conditions, focusing on exotic species and testing EPF genera such as *Beauveria*, *Metarhizium*, *Isaria*, and *Purpureocillium*. However, variations in inoculation methods, environmental conditions, and fungal formulations, have led to diverse results. In addition, the complex biology of these insects, particularly their dependence on symbiotic fungi, represents significant methodological challenges. Field trials (in situ bioassays) are still scarce, and there is a need to move toward standardized protocols and more objective experimental models that consider not only insects’ behavior but also ecological factors. Bridging this gap is essential for successfully implementing EPF-based strategies to assess ambrosia beetles’ biocontrol.

## Simple Summary

Ambrosia beetles are insects that infest stressed or dying trees and cultivate fungus as a food source. Some species, such as *Xyleborus glabratus* and *Euwallacea fornicatus*, may seriously damage fruit and forest trees, including avocado orchards, in addition to spreading harmful fungi that cause tree diseases. This review explores the use of naturally occurring fungi that infect insects, called entomopathogenic fungi (EPF), as an environmental-friendly approach to control these beetles. Although many of these fungi are effective in laboratory bioassays, their effect under real-life conditions, such as in infected orchards or forest trees, is still unknown. Studies must also consider that these beetles depend on their symbiotic fungi for survival. This review will focus on bioassays to assess the effectiveness of these fungi under field conditions. Adopting fungi as natural control strategies will help protect our forest and orchard ecosystems and reduce dependence on chemical pesticides.

## 1. Introduction

Ambrosia beetles are a group of wood-boring beetles belonging to the subfamily *Scolytinae* [1]. They are characterized by their symbiotic relationship with microorganisms, particularly fungi, which are cultivated by female beetle on the walls of the galleries that they construct inside the host trees, serving as a food source for the beetles and their developing larvae [1,2]. Globally, approximately 6000 species within this subfamily are known, most of which play a key role in the wood decomposition within forest ecosystems, acting as primary colonizers of dead or weakened trees [3]. Among this group, around 50 species—mostly exotic—have the potential to attack and infect living trees. Beetles and symbiotic fungi cause significant damage to tree trunks, leading to their death, thus becoming an important phytosanitary threat. In tropical and subtropical regions, ambrosia beetles have been associated with damage to urban green areas and fruit trees such as avocado [4,5,6].

In forest and orchard ecosystems, the use of chemical products for insect pests and vectors control is strictly regulated. Preventive treatments like bifenthrin and permethrin have shown effectiveness in reducing beetle population and preventing new infestations [7]. Although often effective, their application may have negative consequences on human health, non-target organisms, and the commercial value of wood and fruit products, thereby limiting their international trade [8,9,10,11]. Consequently, the management of ambrosia beetles has primarily focused on preventive strategies, involving continuous monitoring, along with cultural practices and semiochemical methods [12,13]. In recent years, there has also been growing interest in the potential of biological control agents, particularly parasitoids and entomopathogenic fungi (EPF) [14,15,16,17].

The use of EPF may be a viable initial alternative. Numerous studies have demonstrated their effectiveness under laboratory conditions [18,19]. However, it is essential to interpret these results with caution, as many were conducted under conditions that may not reflect real wood/gallery environments, often overlooking the beetles’ cryptic biology and nutritional needs [1,2].

This review provides an overview of bioassays used to evaluate entomopathogenic fungi (EPF) as biological control agents against ambrosia beetles, with a focus on assessing fungal pathogenicity and virulence. It primarily describes ambrosia beetles, excluding Platypodinae and Scolytinae species, considered true bark beetles (*sensu stricto*), such as *Dendroctonus* and *Ips*. References to these groups are included only when relevant for comparison or broader ecological or methodological context [20].

## 2. Entomopathogenic Fungi

**Figure 1:**
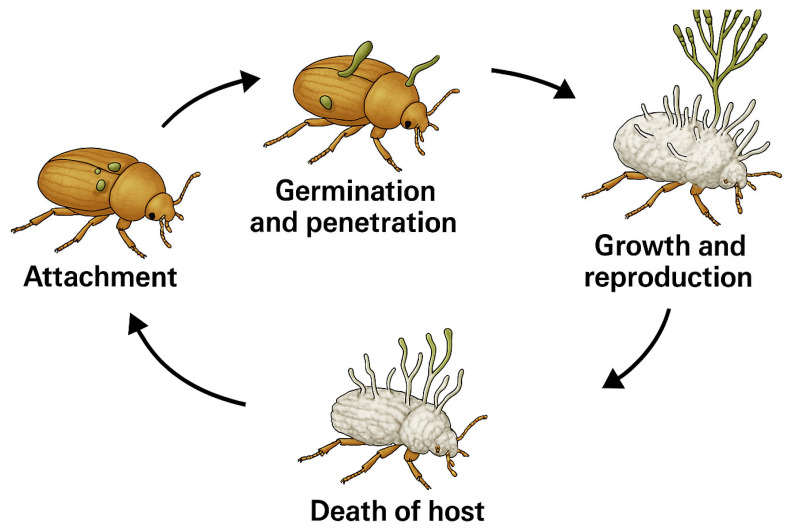
Infectious cycle of entomopathogenic fungi.

These microorganisms have emerged as important biological control agents against various insect pest species, particularly agriculture ones, such as aphids, thrips, whiteflies, and caterpillars, offering an ecological alternative to chemical pesticides [21]. In some cases, they exert minimal impact on non-target organisms and are compatible with other integrated pest management strategies [22,23]. These microorganisms primarily infect insects through their cuticle, penetrating into the hemolymph, where they produce toxic metabolites such as destruxins (produced by *Metarhizium* spp.) or beauvericin (produced by *Beauveria* spp.), that ultimately lead to host death [24,25]. In general, the infection cycle concludes with insect mycosis and the formation of conidia on the host surface, thus facilitating their dissemination and subsequent infection of other susceptible hosts (Figure 1).

The success of insect infection by EPF depends on a variety of biotic and abiotic factors. Relative humidity is critical for the development of infection and disease progression, as well as in the aerial sporulation on insect cadavers. Temperature significantly influences both fungal infection and persistence [26,27] and UV radiation plays a significant role in the depletion and inactivation of fungal propagules in epigeal habitats [28]. Biotic factors such as fungal species, spore density, host plant chemistry, and insect host species also affect virulence and pathogenicity [26].

More than 750 species of EPF have been identified, including *Beauveria bassiana*, *Metarhizium anisopliae*, and *Isaria fumosorosea*, which are commonly used in commercial mycoinsecticides [29,30]. Despite their effectiveness, challenges remain in expanding commercial applications, including enhanced persistence and performance under adverse environmental conditions, such as high temperatures, low humidity, and ultraviolet radiation [31]. Ongoing research focuses on increasing the efficacy and formulation of EPF, as well as understanding their interactions with insect pests, to fully harness their potential in sustainable pest management programs [23,32].

## 3. Ambrosia Beetles

Approximately 3500 species of ambrosia beetles have been described worldwide to date [33]. Their defining feature is an obligate symbiotic association with fungi, a relationship that has independently evolved across multiple lineages, which confers a crucial ecological role as recyclers of organic matter (organic matter producers) [34,35]. The beetles excavate galleries in weakened, dying, or dead trees, where they cultivate symbiotic fungi, which are carried in internal or external specialized structures, known as mycangia. These fungi are the main nutritional resource for adults and larvae [36,37]. In some cases, the fungi obstruct the vascular system of healthy trees, leading to severe damage in forest and orchard ecosystems [38,39]. Several invasive species, such as members of the *Euwallacea fornicatus* complex and the genus *Xylosandrus*, have become established in several regions, including Europe, Argentina, and the United States, where they have caused severe damage to multiple tree species [40,41,42].

Many Scolytine beetles are highly mobile and invasive, with at least 163 species identified as having a cosmopolitan distribution [43]. Their global spread is facilitated by international trade in wood products and live plants, as well as by global warming [12,44,45]. One of the most recent and notable cases is the South Asia native redbay ambrosia beetle (*Xyleborus glabratus*), which has become a major invasive pest in the United States since its detection in 2002 near Savannah, GA, USA, and rapidly spread throughout the southeastern United States, reaching Florida and becoming established in six other states by 2013 [46]. In 2014, its presence was documented in Louisiana, marking its first appearance west of the Mississippi river [47]. This beetle is a vector of the pathogenic fungus *Raffaelea lauricola*, which causes laurel wilt disease in the Lauraceae family trees, including avocados [6,48]. It has spread across 12 southeastern U.S. states, killing millions of trees [49]. Its presence on the continent poses a serious threat to countries like Mexico, the world’s leading avocado exporter, which also harbors a significant diversity of Lauraceae species [50]. Moreover, native beetles such as *Xyleborus affinis*, although not currently considered a phytosanitary issue, have been reported in some studies colonizing avocado trees in Mexican orchards. They also possess the potential to act as vector of *R. lauricola* [51,52,53,54].

## 4. Entomopathogenic Fungi Against Ambrosia Beetles

In recent years, research on EPF as biological control agents against ambrosia beetles has gained attention, primarily driven by the economic impact posed by various invasive and native species, as mentioned above. Table 1 provides a current representative summary of the most relevant studies regarding the application of EPF to this group of insects.

The most frequently used fungi belong to the genera *Beauveria* and *Metarhizium*, with *Isaria* and *Purpureocillium* (=*Paecilomyces*) used to a lesser extent. Some of the strains evaluated originated from reference and local collections [18,55,59,60,61,63,65], and others were obtained from commercial products [19,56,57]. Furthermore, the majority of strains were isolated from non-scolytine insects [56,57,58,59,63], with only two studies involving strains isolated from scolytine beetles [60,65].

A detailed analysis of the available data reveals that all trials have been exclusively conducted under laboratory conditions. This methodological limitation has been widely recognized as a key barrier to applying biological control in practice. It highlights the urgent need for field-based evaluations, to develop more realistic and effective application strategies [26]. Moreover, current studies have primarily focused on exotic pest species, such as *Xylosandrus crassiusculus*, *X. glabratus*, and *E. fornicatus*. Three species—*X. affinis*, *Xyleborus bispinatus*, and *Xyleborus volvulus*—included in some trials, are not considered a high-risk species, which highlights a research gap regarding native or economically inconspicuous species that have the potential to vector phytopathogenic fungi [50,52].

Another noteworthy aspect is the considerable variability in EPF effectiveness, reported across studies. This variability can be explained by multiple factors. First, the susceptibility of the target insect widely varies among species and even between populations, which may be related to genetic, physiological, or ecological differences [66,67,68]. Second, the pathogenicity and virulence of the fungi differ among genera, species, and strains, influencing the host’s response to the microorganism [68,69]. Moreover, fungal formulations—whether as dry spores, emulsions, or commercial preparations—may significantly affect efficacy, particularly due to differences in fungal adhesion to the insect cuticle [24,56]. Furthermore, the methodological diversity of laboratory trials makes it difficult to compare results across studies. Variables such as inoculation method and post-inoculation conditions significantly vary between experiments.

## 5. Bioassay Systems

A bioassay is an essential method in the evaluation of EPF for insect control, serving as one of the most critical stages in the development and eventual commercialization of biopesticides [70]. Bioassays allow for the verification of the fungal agents’ effectiveness against the target pests, which is fundamental for validating their practical application [71,72].

In vivo bioassays, typically conducted under laboratory conditions, aim to assess the pathogenicity and virulence of fungal strains against target insects in a controlled environment, which ensures replicability and variable control [73]. In contrast, in situ bioassays are performed in the host insect’s natural environment, allowing researchers to observe fungal efficacy under real-world conditions, with reduced experimental controls and increased environmental variability [74,75].

Studies have shown that the efficacy of EPF considerably differs across experimental settings. Under laboratory conditions, although efficacy varies depending on the insect species, mortality rates commonly ranges between 80% and 100%. However, in field conditions, effectiveness tends to significantly decline, with reported mortality rates ranging from 28% to 82% [76,77,78]. This reduction is primarily associated with environmental factors such as ultraviolet radiation, temperature, and humidity, which may substantially influence the activity and persistence of EPF in natural settings [78,79].

### 5.1. In Vivo Bioassays on Ambrosia Beetles

When assessing the efficacy of an EPF against ambrosia beetles, it is essential to consider the following key elements: (1) the origin of the beetles used in the assays, (2) the method of inoculation, and (3) the bioassay conditions under which the insects are maintained, following inoculation.

#### 5.1.1. Origin of Beetles

For laboratory assays aimed at evaluating the efficacy of EPF in ambrosia beetles, two main approaches have been used to obtain the insects: (1) direct collection from infested host plants in the field [56,57,59,60,65] and (2) the establishment of laboratory colonies using artificial diets [18,19,55,57,58,61,62,63,64]. The first approach, although frequently used due to its initial practicality, has important limitations, including the lack of control over critical variables such as quantity, physiological state, age, and health of the collected individuals [20]. Moreover, beetles obtained from natural environments are likely to carry microorganisms or natural enemies—such as bacteria, fungi, or nematodes—that may act as confounding factors in mortality assays and compromise the validity of the results [80,81]. In this regard, although some studies with ambrosia beetles have addressed this issue through molecular analyses to rule out latent infections or microbial contamination [56], this is not yet a widespread practice. Nevertheless, it should be considered a standard measure when using field-collected individuals, especially in studies focused on assessing the pathogenicity or virulence of fungal strains.

On the other hand, the use of artificial diets for rearing ambrosia beetles in the laboratory represents a more robust alternative, and is therefore widely employed [70,82,83,84,85,86,87]. This strategy allows for the maintenance of controlled populations, ensures homogeneous age among the individuals used, and significantly reduces the risk of contamination by external agents. It also facilitates the replication of experiments and the comparison between treatments under standardized conditions, which is crucial for the scientific validity of the results [70]. However, not all ambrosia beetle species easily adapt to rearing on artificial diets [87], which presents an additional challenge for its widespread implementation.

#### 5.1.2. Inoculation Methods

In laboratory studies focused on evaluating the pathogenicity and virulence of EPF against ambrosia beetles, various inoculation methodologies have been employed [88]. One of the most used methods, due to its simplicity, reproducibility, and effectiveness, is the immersion of insects in suspensions with known concentrations of conidia [19,56,61,62,64]. This technique enables uniform contact with the inoculum and typically results in a high fungal load on the insect’s body surface.

Another documented strategy involves the direct application of the inoculum using manual sprayers or pneumatic devices, such as the Potter spray tower [18,19,55,58,60,64]. In addition, indirect inoculation methods have been implemented, which consist of depositing the inoculum on surfaces such as filter paper or plant material, over which the insects are allowed to walk for a specific period [18,19,57,58,59,63].

Each of these strategies involves differences in the number of conidia that adhere to the insect’s body. Immersion typically results in a significantly higher fungal load, with the possibility that conidia may penetrate cavities such as spiracles, the mouth, or the anus, potentially accelerating systemic infection [89]. In contrast, indirect methods generally result in a lower initial load, typically limited to regions such as the legs or the ventral surface [56,63]. However, factors such as the duration of exposure to the inoculated surface can significantly influence the amount of inoculum that adheres [19]. Interestingly, comparative studies between direct and indirect methods have not shown significant differences in terms of beetle mortality or survival time [59,60,64], suggesting that fungal effectiveness may depend more on its intrinsic virulence than on the initial quantity of conidia.

Despite the practicality of direct inoculation strategies, their results should be interpreted with caution, especially when aiming to extrapolate them to field conditions. The application of EPF in forest systems or agroecosystems with woody plants presents considerable challenges in terms of coverage and efficacy [90]. Unlike low-growing herbaceous crops, where target insects—often sessile—are almost fully covered by applications, achieving effective contact between the pathogen and the host. In contrast, the use of EPF is difficult in trees [91,92]. In these settings, applications are typically limited to trunks or canopies through spraying, where the likelihood of reaching the beetles—generally found inside galleries—is low [93]. In this context, bioassays using indirect inoculations may offer a more realistic model of EPF performance under natural conditions, as they more accurately simulate the type of contact that occurs in the field. Therefore, despite their methodological complexity, such approaches should be promoted and generalized in future research.

Among the indirect inoculation methods, filter paper has been occasionally used in studies with ambrosia beetles [19,57,63,64]. Although it is easy to manage and widely accessible, its high absorbent potential may reduce the availability of conidia on the surface, thereby decreasing the efficacy of insect contact and sometimes yielding inconsistent results [26,64]. Alternatively, the use of plant material such as branches, bark, or logs has proven to be a more realistic option that mimics the insect’s natural environment and has been successfully used in numerous studies [19,55,59,60,65]. In these assays, reported mortality rates have ranged from 60 to 100%, highlighting the potential of EPF under exposure conditions that more closely resemble those in the field.

### 5.2. Post-Inoculation Conditions

As mentioned above, ambrosia beetles are insects with an obligate symbiotic relationship. They are considered “farmer” beetles because they cultivate the fungi that serve as their food source during both adult and immature stages [94]. Bioassays conducted with this type of insect must undoubtedly take this mutualistic relationship into account. In our personal experience, in the case of *X. affinis*, these insects do not survive for more than five days in humid chambers when isolated (unpublished data). Therefore, at least for this species, it is not possible to maintain beetles without their food source. The same may be expected for other ambrosia beetle species. A considerable portion of the reported studies consistently disregard the insect’s feeding, keeping them under starvation conditions in humid chambers after being directly inoculated [18,19,55,56,59,60,65], or maintaining them throughout the trial period in contact with the filter paper on which the inoculum was placed [57,58]. These studies report low mortality in control groups, among different beetles, suggesting that starvation was not a significant factor and raising the possibility of a species-specific tolerance to food deprivation.

The relationship between insect starvation and their susceptibility to EPF has been marginally studied. However, available evidence suggests that lack of food significantly influences such susceptibility. Nutritional stress, including food deprivation and poor diet, has been shown to increase the vulnerability of several insect species to *B. bassiana* [95,96].

One way to counteract the effects of food deprivation in ambrosia beetles during bioassays is through the use of plant material, such as branches or logs, or by providing artificial diets that support the cultivation of their symbiotic fungi [96]. In indirect inoculation assays using branches or logs, it is implicitly assumed that the substrate serves for gallery formation, which nourishes the insect.

Based on the experience of our research group, we have concluded that the mere contact between the insect and a plant substrate does not necessarily guarantee colonization. It is common for branches or logs to be placed inside containers, which increases temperature, humidity, and the concentration of volatile compounds. These conditions often induce erratic behavior in the beetles, which tend to focus more on escaping the container than on actively boring into the substrate. In such cases, beetles often make only superficial perforations in the bark without developing internal galleries. As a result, they are deprived of food and use a considerable amount of energy, chaotically wandering over the surface. This phenomenon must be considered to avoid misinterpreting the lack of boring or gallery formation as a potential effect of EPF infection. One method that might help minimize this behavior is to soak the plant material with ethanol, a simple chemical compound to which ambrosia beetles respond [18,97]. Recently, methodological strategies have been implemented in which beetles are placed in small tubes attached directly to the surface of the log, allowing for direct and controlled contact with the substrate [65]. The issues described above are less common when artificial diets are used; although, as previously noted, this depends on the species under study and the specific formulation of the diet [86,87]. Taking this into consideration, parameters such as boring activity, gallery formation, and progeny are highly informative. In studies that have evaluated boring activity, rates have ranged from 28% to 83% in natural branches and from 65% to 95% in artificial diets [24,56,98]. Regarding progeny, reductions of up to 97% have been documented [55,65].

A crucial factor to consider when using natural or artificial diets in bioassays is the removal of conidia during gallery formation by the insect. This variable has been appropriately considered by some authors, who in their bioassays chose to keep insects in humid chambers after inoculation and before placing them onto the substrate, thereby avoiding conidial removal [55]. This strategy may be advantageous for the fungal infection process and represents a critical point to consider when interpreting and designing bioassays involving ambrosia beetles. Among the few studies addressing conidial removal, it is estimated that boring activity may remove more than 70% of the conidia in about 12 h in artificial diet settings [63], which is a significant amount, especially considering that the number of infective units adhered to the insect depends on the inoculation method, as mentioned above. Therefore, to achieve higher mortality rates, it is necessary to apply higher doses in such a way that the insect acquires enough infective units, thereby counteracting the removal effect during gallery formation. This has been observed in beetles such as *X. affinis*, where immersion inoculation with *B. bassiana* required a dose of 1 × 10^9^ conidia/mL to achieve mortality rates slightly above 50% [63], when beetles were released onto artificial diet after inoculation.

### 5.3. In Situ Bioassays on Ambrosia Beetles

To date, there is no evidence of field evaluations using EPF for the control of ambrosia beetles. However, the accumulated experience from studies conducted on bark beetles of the genera *Dendroctonus*, *Ips*, and *Pityophthorus* provides a useful framework for proposing methodological guidelines that facilitate the design and implementation of similar trials for ambrosia beetles.

Field trials have yielded mixed results. Some studies report a significant reduction in tree mortality and bark beetle infestations [98,99,100,101]; others have observed limited or no effects under similar conditions [74,102,103]. The variability in efficacy is likely influenced by environmental factors such as ambient temperature, ultraviolet radiation, and phytochemicals produced by host trees, all of which may affect the viability and infectivity of entomopathogenic fungi [20]. Moreover, for fungal applications to be effective, it is critical to consider the complex ecological interactions between bark beetles and their environment, including their symbiotic associations and specific habitat characteristics [104]. The development of specialized formulations or delivery systems has shown promising results in improving infection success in natural environments, particularly in species such as *Scolytus amygdali* and *Dendroctonus ponderosae* [105,106].

Among the most effective experimental approaches are the spraying of log sections—commonly referred to as trap logs—under field conditions [74,99,102,107], and the use of autodissemination traps baited with pheromones to enhance beetle attraction and contact with the pathogen [100,101,103,108]. In addition, trials conducted in controlled environments have tested the release of beetles previously inoculated with entomopathogenic fungi, offering insights into the pathogen’s transmission potential [100,101]. Nonetheless, some researchers have raised concerns about the extrapolation of these laboratory or semi-natural results to real-world conditions, citing limited environmental representativeness and potential overestimation of field efficacy.

## 6. Future Directions

Despite the notable progress in evaluating EPF against ambrosia beetles under laboratory conditions, their practical implementation still faces significant challenges. One of the key future priorities should be the transition toward field-based bioassays that allow for validation of laboratory-observed efficacy under real environmental variables such as humidity, temperature, and UV radiation, all of which directly influence the persistence and activity of EPF [31,32,33,78].

It is also necessary to standardize inoculation methods and post-inoculation conditions, as current strategies exhibit high variability in terms of mortality, gallery formation, and conidia removal [55,56,63,64]. It is recommended to increase the use of plant material as a substrate in bioassays to more realistically replicate natural conditions [55,59,60,65], as well as to explore new formulations that enhance conidial adherence and persistence on the insect cuticle [24,56].

Taken together, several EPF species, particularly *Beauveria bassiana* and *Metarhizium brunneum*, have shown significant potential to control ambrosia beetles. However, their effectiveness may vary depending on environmental conditions, target species, and the specific fungal strain used. Therefore, further testing under field conditions is essential to accurately selecting strains for this pest management tool assessment.

Furthermore, the inclusion of native beetle species in future studies is essential, given their potential to become significant pests and act as vectors of pathogens [9,61,62,63]. Addressing these aspects will be key to advancing toward sustainable and effective biological control strategies for these insects.

## Figures and Tables

**Table 1:** Studies developed with entomopathogenic fungi for the control of ambrosia beetles.

Fungal Species/Strain	Target Species	Efficacy Level	Inoculation	Post-Inoculation Conditions
*Metarhizium brunneum* F52, *Beauveria bassiana* Naturalist, *B. bassiana* GHA	*Xylosandrus germanus*	6.7–61.7% [55]	Direct	Wet chamber/Artificial diet
*M. brunneum* F52, *B. bassiana* Naturalist, *B. bassiana* GHA	*Xylosandrus crassiusculus*	76.7–95.6% [18]	Direct and Indirect	Wet chamber/ Natural diet
*Isaria fumosorosea Ifr*, *I. fumosorosea PFR*, *B. bassiana GHA*	*Xyleborus glabratus*	54.7–77.3% [56]	Direct and Indirect	Wet chamber/Natural diet
*M. brunneum* Met52^®^, *I. fumosorosea* PFR-97^®^, *B. bassiana* GHA(BotaniGard^®^)	*Xyleborus bispinatus* *X. crassiusculus* *Xyleborus volvulus*	Unspecified [57]	Indirect	Wet chamber
*Purpureocillium lilacinum* TR1	*X. germanus* *Xyleborus dispar*	94.7–100% [58]	Direct	Natural diet
*I. fumosorosea* TR-78-3	*Anisandrus dispar* *X. germanus*	90–100% [59]	Direct and Indirect	Wet chamber
*M. anisopliae* TR-106, *B. bassiana* TR-217	*X. germanus*	64–100% [60]	Direct and Indirect	Wet chamber
*B. bassiana* CNRCB-CHE 44, 171, 431 and 485	*Xyleborus affinis*	40–58.7% [61]	Direct	Artificial diet
*Metarhizium robertsii* Xoch 8.1	*X. affinis*	76% [62]	Direct	Artificial diet
*B. bassiana* CNRCB-CHE 44, 171, 431 and 485	*X. affinis*	3.4–50.3% [63]	Direct	Artificial diet
*M. anisopliae* + *B. bassiana* (Bio-Insek^®^), *B. bassiana* (Eco Bb^®^)	*Euwallacea fornicatus*	Unspecified [19]	Direct and Indirect	Wet chamber/ Natural diet
*B. bassiana* CNRCB-CHE 44, and 485 strains	*X. affinis*	10–20% [64]	Indirect	Artificial diet
*B. bassiana* B-BB1	*Euwallacea interjectus*	64–100% [65]	Direct and Indirect	Wet chamber/ Artificial diet/ Natural diet

## Data Availability

No new data were created or analyzed in this study. Data sharing is not applicable to this article.
